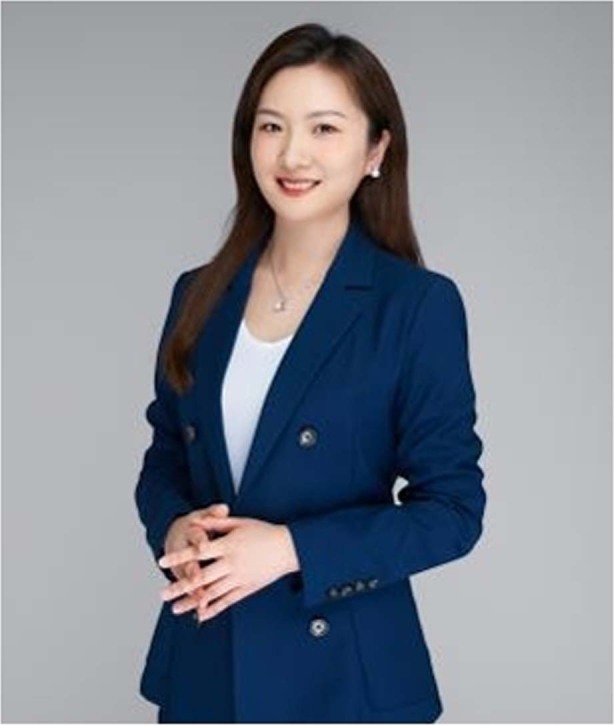# Light People: Professor Byoungho Lee

**DOI:** 10.1038/s41377-021-00683-7

**Published:** 2022-01-01

**Authors:** Hui Wang, Cun Yu

**Affiliations:** Department of International Cooperation, Changchun Institute of Optics, Fine Mechanics and Physics, Chinese Academy of Sciences, 3888 Dong Nan Hu Road, 130033 Changchun, China

**Keywords:** Microscopy, Inorganic LEDs

## Abstract

Major developments were made recently in both VR (virtual reality) and AR (augmented reality) technologies, which became the focus of attention. In recent years, MR (mixed reality) technology has also emerged, and optical components play an irreplaceable role in these technologies.

Professor Byoungho Lee, who graduated from the University of California at Berkeley and currently works at Seoul National University in South Korea, has been committed to the development of optical components used in VR and AR technologies. As a pioneer of optical electronics in Korea, he is involved in various well-known academic organizations in the optical field, such as the Optica, SPIE, and IEEE, as well as serving as the president of the Optical Society of Korea, leading the direction of the development of optical industry in Korea. As the ambassador of China-Korea Optoelectronics Exchange, Prof. Lee has also played an active role in Chinese optical events and activities. Over the years, he and the Journal *Light: Science & Applications* (LIGHT) have made progress together and have both made their names in the vast field of optoelectronics.

So where did the story between Prof. Lee and the LIGHT journal begin? And what kind of link does the professor have with Changchun Institute of Optics, Fine Mechanics and Physics (CIOMP)? How did he become a pioneer in optoelectronics technology? These are the questions we are eager to ask Prof. Byoungho Lee.

The future cannot be predicted, but it can be invented, said Dennis Gabor who had invented holography. The pace of human technological advancements has never stopped. Who is to say that we cannot take a virtual tour of the Palace Museum or explore the north and south poles in the future? Scientists like Prof. Lee are working hard to use technology to provide mankind with an intelligent lifestyle, and lead a new technological trend. I am sure we are all looking forward to it.

## Abstract (web summary)

As a pioneer of optical electronics in Korea, Professor Byoungho Lee has been committed to the development of optical components used in VR and AR technologies.

## Graphical abstract

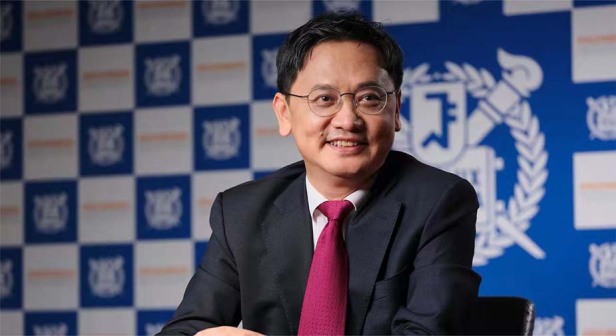

**Biography:** Byoungho Lee received his B.S. (1987) and M.S. (1989) degrees at Seoul National University and Ph.D. degree in Electrical Engineering and Computer Sciences at the University of California at Berkeley in 1993. He joined Seoul National University as a faculty member in September 1994 and is currently serving as the Dean of College of Engineering of the university. Prof. Lee is a Fellow of SPIE, OSA (Optica), IEEE, and the Society for Information Display (SID). He is also a (Senior) Member of the National Academy of Engineering of Korea and the Korean Academy of Science and Technology (KAST). He is, especially, in charge of managing the young scientists group of KAST (Y-KAST). He has served as the President of the Optical Society of Korea and now is serving as the president of the Korean Information Display Society. His research fields are 3D display, AR/VR display, and metasurface devices. He has received many awards including the National Science Badge of Jinbo-Jang of Korea and the Special Recognition Award of the SID.

**Figure:**
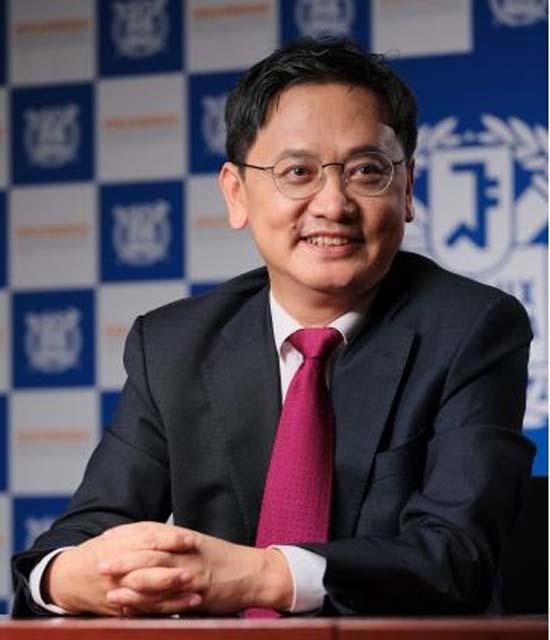

**1. You and your team are developing advanced head-mounted displays for three-dimensional augmented reality, light field displays, and real-time three-dimensional microscopy. What do you think are the future development direction and application fields of 3D technology?**

With the recent interest in metaverse, more attention on optical devices for augment reality (AR) and virtual reality (VR) are being payed. For AR and VR head-mounted or near-eye displays, because images are provided to both eyes in general, 3D effect is becoming important. 3D televisions were not successful in the past due to the cost and lack of 3D contents. However for AR/VR eye-wear systems, providing stereo images is essential and so, 3D display becomes unavoidable. In that sense, more research and development for 3D technology is needed.

**2. In recent years, new technologies such as VR (virtual reality) and AR (augmented reality) have received much attention from the public. You have been engaged in more high functioning optical elements such as lenses, polarizers, and spatial light modulators based on metasurfaces and holographic optical elements. What role do optical elements play in VR and AR technology? What are the current challenges?**

VR and AR systems require a kind of integration of many technologies from optics to sensors and image rendering. But the optics is a key in such devices. There are several important parameters such as eye-box size, resolution, field of view, focus cues, and form factor. There is a trade-off relationship among those parameters. To optimize the system with good performance, the display such as micro OLED and micro LED is important and the designing optics system is the most important part. To minimize eye-fatigue and reduce the volume and weight of the eye-wear system is the key player for the AR/VR systems to be really successfully commercialized.

**3. In 2018, you were invited to participate in Optics Frontier―The 10**^**th**^ **International Conference on Information Optics and Photonics (CIOP 2018), and introduced the use of digital holography and machine learning in the capturing and separating of overlapping fingerprints, which has the advantage of being a non-destructive detection method. Can you tell us more about this technology please? Three years on, what are the latest achievements and progress of this technology?**

We made two systems. One is the digital holographic microscopy system that detects the 3D profile of fingerprint images. General commercialized digital holographic microscopy systems cannot be applied for fingerprint measurement because their capturing area is much smaller than the size of fingerprints. So, we developed a system that fits to our purpose. Another is to remove background noise and separate overlapped fingerprints by using the deep learning algorithm. The systems were very successful and we recently transferred our technologies to the Korean National Police Agency.

**4. As an ambassador for China-Korea optoelectronics exchanges, you have been actively involved with various Chinese academic organizations: China Optical Engineering Society, China Optical and Optoelectronics Manufactures Association, China Physical Society, as well as universities, research institutes, and other international organizations. Why are you so keen to participate in such activities? How do you evaluate the development of China’s optoelectronic industry?**

China has been rapidly growing both in research and technology development in optics, display, and photonics. In some areas, China has become world-leading key player. So China is an important county in my areas of optics and display technologies. Many professors in China have made venture companies to transfer their lab technologies to real fields, which is also very important and demanding in the society in these days.

**5. You are a Fellow of IEEE, OSA, SPIE, and SID, and a Member of the Korean Academy of Science and Technology, etc. What do you think you have gained from participating in these international academic organizations?**

I have served on the Board of Directors of OSA (recently changed its name to Optica) and is serving on the Board of Directors of SPIE. When I serve for the positions, I try to work to be helpful for the societies themselves. But on another hand, I have learned a lot from the procedure of strategic planning process and decision-making process of such huge well-organized international societies. The lesson was useful when I served as the President of the Optical Society of Korea and the President of the Korean Information Display Society. Currently, I am serving as the Dean of College of Engineering of Seoul National University. My international academic experience is also quite helpful in doing my job as the dean.

**6. You were the editor-in-chief of the Journal of the Optical Society of Korea, and the Journal of Information Display. In addition, you were also on the editorial board of** ***Light: Science & Applications*** **(LIGHT)**, ***Advances in Optics and Photonics***, ***Optics Letters***, ***Applied Optics***, ***Journal of the Society for Information Display***, ***Japanese Journal of Applied Physics*, and** ***Applied Physics B*. What do you think are the similarities and differences in the development of journals in different countries?**

To serve on the editorial board of journals are all similar. Of course, different journals have different impact factors. Editorial boards of many journals are keen to journal ranking or impact factor. There is competition among journals. The right way to pursue for each journal is to attract high-quality papers and serve for the community. Some China-based journals such as *Light: Science & Applications* and *Photonics Research* are high-profile journals. For professors to publish in high-impact journals becomes more important in these days for promotion and getting big government research fund. There are so many journals. So, it is important for journals to become journals that attract many readers and high-quality papers.

**7. As a former editorial board member of** ***Light: Science & Applications*** **(LIGHT), can you talk about your relationship with the Journal? Do you have any expectations and suggestions for LIGHT?**

I was on the editorial board of LIGHT from the birth of the journal and I quit it a few years ago. That was because I thought it is important for the journal to recruit new young outstanding scholars on the editorial board. LIGHT is doing well. But I hope it could deal with more diverse topics and the review period needs to be further shortened. It is good for the journal to have a new sister journal *Light: Advanced Manufacturing*.

**8. You have participated in the Light Conference organized by Light Publishing Group many times. What do you think of the conference? Any suggestions?**

It is an excellent high-quality conference series. My suggestion to improve the conference is to attract contributed poster presentations outside of China as well. Because the keynote and invited speakers are very distinguished scholars, if non-Chinese students could attend the conference it will be good for them and also CIOMP in promoting its research reputation.

**9. You and your research team have published 400+ peer-reviewed international journal papers, 700+ international conference papers, and 20+ books and chapters of books. Your accumulated citation reaches over 14,000 and h-index is 62. Do you have any tips or advice for students and young researchers on how to write a high-quality academic paper?**

It is needed to make graduate students have ambition that they want to do world top research and compete with world top research groups in their fields. Well organizing of the labs in such a way that senior graduate students train junior graduate students both in theory and experiment is also important. If the laboratory is too personalized, knowledge and know-how of previous students are not delivered to new students. Collaboration with other groups with open mind is also necessary for high-quality synergy research. In general students and scholars are very keen on the first authorship and corresponding authorship. Fair decision regarding the authorship is important for continuing collaborative research.

**10. You have received many distinguished academic awards such as the Jinbo-Jang National Science Medal of Korea, the Special Recognition Award of the Society for Information Display, the Grand Academic Award of the Optical Society of Korea, etc. How do you feel about these honors?**

I really appreciate all the awards and am proud of them. I have served on many awards committees including the Award Committee of Board of Directors of OSA. For good awards, there are so many highly qualified candidates. Even if one is not successful in receiving any award, one needs not to be depressed. In many cases, to be included in the final list of candidates is an honor itself. If one is not in the list of candidates, he or she does not have any chance to be awarded. In that sense, it is better for someone to nominate you or you apply for awards in some cases. But too many applications might not be so good. It does not give good impression to evaluation committee members.

**11. For the 60**^**th**^ **anniversary of the establishment of the Changchun Institute of Optics, Fine Mechanics and Physics, Chinese Academy of Sciences (CIOMP), you were invited to attend the Photonics Trend 2012 International Conference that year. In 2018, at the invitation of the OSA/SPIE Student Branch of CIOMP, you visited CIOMP again. What is your impression of CIOMP?**

With several visits I became to know about the CIOMP quite well. CIOMP is the first research institute in optics established in China. I have witnessed many eager researchers and students of CIOMP. Optics and photonics have diverse areas from fundamental research to applications. My understanding is that CIOMP is a very outstanding institute especially in the research of optical engineering and applications.

**Figure 1:**
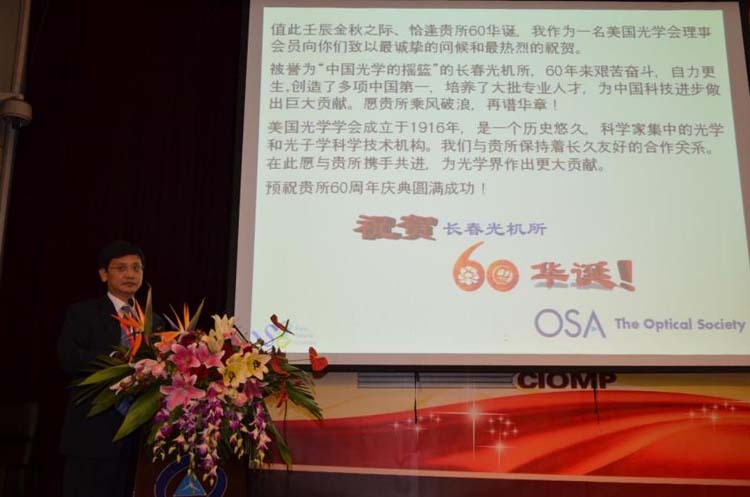
Prof. Lee gave a talk on the international conference for celebrating CIOMP’s 60^th^ Anniversary.

**Figure 2:**
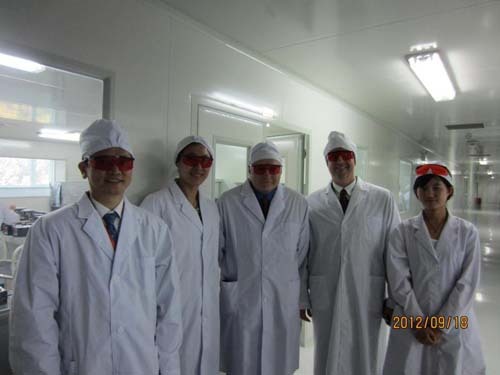
Prof. Lee, as a member of the OSA delegation, visited the Changchun New Industries Optoelectronics Technology Co., Ltd. based at CIOMP.

**12. COVID-19 has changed our lives greatly. Many scientific research projects have been interrupted or delayed. Online international conferences have become the new norm. Personally, do you prefer online conferences or offline? Could you give some suggestions or advice to help researchers go through this unusually tough period?**

International optical academic societies have evaluated the online conferences that were held after the COVID-19 situation. In fact, online conferences were not so successful. Although many people have registered for the conferences, number of views for each presentation video was below expectation in general except for distinguished invited talks. To my understanding, online exhibitions were not so successful either. For online international conferences, because registered people access the conferences from their countries at work or at home, their attention is distracted. Another factor is to build up human relationship and network, which can be hardly done by online conferences. In US most conferences are now being converted to the format of offline conferences or hybrid conferences. Although online conferences are not so successful generally speaking, I advise the researchers to take a full advantage of online conferences by watching good talks. Differently from offline conferences, you can look at the presentation slides in detail.

**13. You received your Ph.D. degree from the Department of Electrical Engineering and Computer Science, University of California at Berkeley. What do you think you have gained from your Ph.D. studies? What impact did it have on your subsequent research career?**

I had received my B.S. and Master degrees from Seoul National University and then went to UC Berkeley with the Korean National Scholarship. To study at such a world-leading university with a kind of freely-thinking environment made me grow much not only in knowledge but also in ways of thinking. In these days Seoul National University is doing very well in research and publishing high-quality papers. A rapid growth of the university research level was greatly enabled by the faculty members who had had good experiences at top universities and top research institutes or companies in more advanced countries. Now situation has changed and many top undergraduate students remain at my university as graduate students. Some of my graduate students are recruited by famous ICT companies in US after graduation.

**14. From a student to a scientist, a teacher and an academician, how do you think your attitude toward scientific research has changed over the years?**

As a graduate student my major concern was my project and fulfillment for getting Ph.D. degree. When I became a professor, first it was a great (but happy) burden to set up future research directions and obtain research funds. As time goes by, to perform outstanding research became more important. With serving as the President of the Optical Society of Korea and the President of the Korean Information Display Society, and now as the Dean of College of Engineering, my view became wider and wider and I used to think about what would be future prevailing technologies.

**15. What obstacles or difficulties have you encountered in your research work? How did you overcome these difficulties?**

In some cases, the output of my research projects is not going well as I originally planned. That is because the original plan was too optimistic and ambitious. In those cases, I revisit the original proposal and think about what was not good from the beginning. I discuss with my students about how we can resolve the issues and make progresses.

**16. In your life and career, who has the biggest influence on you and in what way?**

Besides the religion, my wife is the most affective person to me. I discuss many issues and difficulties in my daily life with my wife.

**17. How do you balance your work and family life?**

I work quite much. I read papers at home and work with notebook computer at home as well. But at the same time, I communicate with my wife and two daughters quite well. My family know about my jobs and schedule well. I spend much time with my family for discussion on family issues, give regular rides to my second daughter, and dine together. For that, I reduce my outside activities with other people, especially in weekend.

**18. What advice do you have for young researchers?**

Dr. Dennis Gabor, who was the inventor of the concept of holography, said “The future cannot be predicted, but futures can be invented.” This wording was modified later by several other people. For example, Alan Kay said “The best way to predict the future is to invent it,” Some people can achieve great performance that affect the society much, while some do not. The attitude of enjoying your work, hoping you will make something very advantageous for human being, is good enough for your research life. And the attitude of thinking big is also important for the growth as a scientist, engineer, or academician.

**Light special correspondent**

*Hui Wang is the Deputy Director of the Office of International Cooperation in the Changchun Institute of Optics, Fine Mechanics and Physics (CIOMP), Chinese Academy of Sciences (CAS). She currently works on international communication and cooperation for the CIOMP and was a founding member for the Nature Publishing Group and CIOMP joint journal Light: Science & Applications. She is the founder of “Rose in Science” and has published several articles in Acta Editologica, International Talent, Light: Science & Applications, etc., and was invited to take an interview by SPIE Women in Optics, which was published in 2015*.

**Figure:**
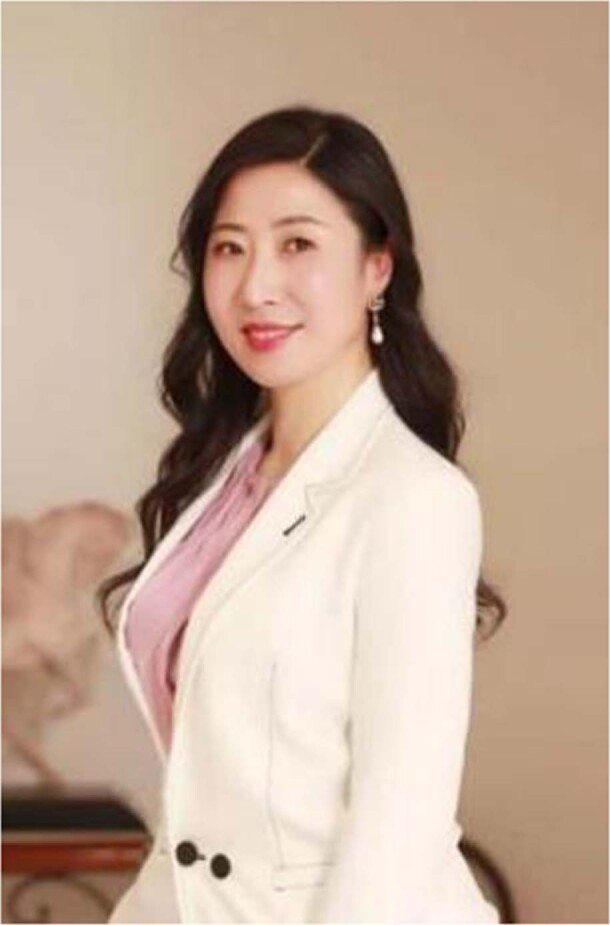

*Cun Yu works at the Office of International Cooperation in the Changchun Institute of Optics, Fine Mechanics and Physics (CIOMP), Chinese Academy of Sciences (CAS). Her main duties cover the Sino-Belarus International Innovation Center, international cooperation projects and exchanges between CIOMP and institutions in Belarus, Ukraine and Russia. She has published multiple articles in the journal International Talent, Light: Science & Applications, and is a member of CSA’s Science & Technology Translations Association*.

**Figure:**